# Cancer: a mirrored room between tumor bulk and tumor microenvironment

**DOI:** 10.1186/s13046-021-02022-5

**Published:** 2021-06-28

**Authors:** Pablo Hernández-Camarero, Elena López-Ruiz, Juan Antonio Marchal, Macarena Perán

**Affiliations:** 1 Department of Health Sciences, University of Jaén, Campus de las Lagunillas SN, E- 23071 Jaén, Spain; 2 Excellence Research Unit “Modeling Nature”: from Nano to Macro (MNat), University of Granada, Granada, Spain; 3 Instituto de Investigación Biosanitaria IBS.GRANADA, E-18071 Granada, Spain; 4 Department of Human Anatomy & Embryology, Faculty of Medicine, University of Granada, Avda. de la Investigación 11, E-18016 Granada, Spain

**Keywords:** Tumor microenvironment, Metastasis, Pre-metastatic niche, Cancer-associated fibroblasts, Tumor-associated macrophages

## Abstract

It has been well documented that the tumor microenvironment (TME) plays a key role in the promotion of drug resistance, the support of tumor progression, invasiveness, metastasis, and even the maintenance of a cancer stem-like phenotype. Here, we reviewed TME formation presenting it as a reflection of a tumor’s own organization during the different stages of tumor development. Interestingly, functionally different groups of stromal cells seem to have specific spatial distributions within the TME that change as the tumor evolves into advanced stage progression which correlates with the fact that cancer stem-like cells (CSCs) are located in the edges of solid tumor masses in advanced tumors.

We also focus on the continuos feedback that is established between a tumor and its surroundings. The “talk” between tumor mass cells and TME stromal cells, marks the evolution of both interlocuting cell types. For instance, the metabolic and functional transformations that stromal cells undergo due to tumor corrupting activity.

Moreover, the molecular basis of metastatic spread is also approached, making special emphasis on the site-specific pre-metastatic niche formation as another reflection of the primary tumor molecular signature.

Finally, several therapeutic approaches targeting primary TME and pre-metastatic niche are suggested. For instance, a systematic analysis of the TME just adjacent to the tumor mass to establish the proportion of myofibroblasts-like cancer-associated fibroblasts (CAFs) which may in turn correspond to stemness and metastases-promotion. Or the implementation of “re-education” therapies consisting of switching tumor-supportive stromal cells into tumor-suppressive ones. In summary, to improve our clinical management of cancer, it is crucial to understand and learn how to manage the close interaction between TME and metastasis.

## Background

Despite the huge effort made by the scientific community in the last century, the incidence of cancer is continuously increasing [1] and it remains an incurable and lethal disease in the majority of cases. It has been widely documented that the tumor microenvironment (TME) of solid malignancies plays a key role in the promotion of tumor progression, invasiveness, metastasis, drug resistance [2–4] and even in the maintenance of the cancer stem-like phenotype [5]. TME is composed of an extracellular matrix, an extensive vascular network, lymphatic vessels, soluble molecules, and cells: cancer-associated fibroblasts (CAFs), tumor-associated macrophages (TAMs), tumor-endothelial cells, pericytes, tumor-associated adipocytes, B lymphocytes or T lymphocytes [6]. It could be believed that CAFs and TAMs have their own behaviour and structural organization as an almost independent entity around the solid tumor mass. However, these stromal cells show heterogeneity based on expression profiles [7, 8], which implies different functional subtypes [9, 10] that may be a direct reflection of the tumor heterogeneity and functional organization during the different stages of the tumor progression timeline.

Interestingly, TME generation and evolution during tumor progression with different stages of CAFs and TAMs functional organization may be related to the model of tumor development described in [11] in which the appearance of cancer stem-like cells (CSCs) in the edges of solid tumor masses is evidence of an advanced stage of the tumor.

Additionally, metastatic growth is considered as the main cause of cancer-related death worldwide although it is not yet a well-understood process [12]. For instance, how a tumor “chooses” the distant metastatic tissue is still a matter of debate. Here, we review the latest progress on metastases spread studies in an attempt to improve our understanding of this controversial process. Furthermore, we analysed consensus observations about the “preference” of some cancers to metastasize in certain tissues such as the bone [13]. The site-specific formation of a pre-metastatic niche, an essential event before metastatic growth, seems to be orchestrated by primary tumor-derived exosomes [14]. In fact, it has been demonstrated that the specific integrins patterns on the exosome surface determine the selection of their cellular targets [15]. Thus, after discussing the latest advances in the understanding of the metastatic spread of cancer, we propose the fundamental role of the changing tumor molecular signature during tumor development, which will be reflected in the molecular profiles of the secreted exosomes, determining the targeted tissues for metastatic nesting. Finally, we argue and suggest the potential clinical implications of an accurate characterization of adjacent to tumor mass TME biopsies and the promising perspectives of determining the most probable target tissue of metastasis. Furthermore, the intrinsic nature of TME formation may give us useful cues to develop more efficient therapeutic strategies.

## Tumor microenvironment: the importance of heterogeneity in CAFs and TAMs

TME of solid malignancies has crucial roles in tumor progression, invasiveness, metastasis, drug resistance [2–4], and even in the maintenance of the cancer stem-like phenotype [5].

The main reported cell subpopulations responsible for heterogeneity within the TME are CAFs and TAMs. Different markers have been postulated for CAFs characterization, such as FAP, PDGFR [16, 17], FSP1, vimentin, fibronectin, αSMA [18] or CD90 [19]. Such a wide range of biomarkers could be explained by both the tumor specific molecular signature and the cell type from which CAFs can originate. In fact, solid tumors are able to promote the transformation into CAF of several cellular types like mesenchymal stem cells (MSCs) [20], fibroblasts [21], or even pericytes [22], and the nature of the precursor cells appears to condition the final molecular profile of the CAF [23].

Considering such a variety of CAF-related markers and that several of these biomarkers could co-exist in a single CAF, it is no wonder that the CAFs classification based on their gene expression profiles show variable CAFs subpopulations between different types of cancers and between different patients with the same tumor type [7, 24]. Additionally, a similar heterogeneity is also observed in proteomics-based studies. For instance, the existence of an inter-patient CAFs heterogeneity considering the functional role of specific LOX/LOXL enzymes secreted by CAFs in different patients with prostate cancer has been revealed [25]. Interestingly, it could be considered that the CAFs heterogeneity is an echo of the inter- and intra-tumor heterogeneity related to differences in DNA mutations, expression patterns and even proteomic profiles intrinsic of each tumor mass.

Tumor cells, of almost all solid malignancies, can be functionally classified into two main groups: well-differentiated/high-proliferative tumor cells and low-differentiated/low-proliferative CSCs. Importantly, such functional classification has been associated with the surrounding TME organization [26]. Indeed, several studies established two functionally different groups of CAFs. For instance, in prostate cancer, it has been shown that a first subgroup of CAFs promoted the proliferation of cancerous cells by secreting Wnt-3a, whereas a second subgroup of CAFs enhanced the metastatic capacity and invasiveness (well-known properties of CSCs) of the cancerous cells by the secretion of SDF1 [27]. Similar findings were observed by Patel and co-workers in oral carcinoma in which a subgroup of CAFs prompted the hyperproliferation of tumor cells whereas another subgroup of CAFs enhanced the stemness and, thus, the metastatic potential [9].

These two functionally distinct CAFs subpopulations can also be identified in other studies as pro-inflammatory CAFs, which may correspond to the proliferation-promoting CAFs [28], and myofibroblasts-like CAFs which may in turn correspond to stemness and metastases-promoting CAFs [29]. Notably, Pelon and colleagues showed αSMA like a global myofibroblasts-like CAFs-associated biomarker. In this context, fibronectin and calponin have also been presented as myofibroblasts-like CAFs biomarkers [30].

In regards to TAMs, a high amount of TAM-related biomarkers including CD68, CD14, HLA-DR, CD163, CD204, CD206, CD209, TREM-1 [31], or MARCO (macrophage receptor with collagenous structure) has been described [32].

TAMs can mainly be derived from circulating monocytes or tissue-resident macrophages and these different origins might condition the specific TAM markers [33], partially contributing to their heterogeneity based on biomarker expression. Moreover, the specific molecular signature of the tumor of origin may also condition the final expression profiles and biomarkers exhibited by TAMs (see following section). Similar to CAFs, a single TAM could also express several of the exposed biomarkers simultaneously, thus, the existence of different TAMs subpopulations between the cancer types and patients with the same malignancy is not surprising [8, 34]. On the other hand, TAMs can also be functionally classified into two main groups: (i) classically activated, tumor-suppressive M1 TAMs, and (ii) alternatively activated, tumor-supportive M2 TAMs [10]. Moreover, it has been shown that M1 macrophages are closely related to inflammation and pathogen killing whereas M2 macrophages are related to tissue repair [35]. It has identified M1-related markers, such as MHC-II, CD40, CD80 or CD86 [36], and M2-related markers including CD163, CD204, CD206 or CD209 [31], although, the existence of TAMs expressing both M1 and M2 related markers has also been reported [37–39] increasing the high heterogeneity described for TAMs and making it difficult to make a neat classification.

Interestingly, it has been reported that both CAFs and TAMs display specific spatial distributions within the TME and tumor mass, which could be related to the spatial distribution of CSCs mainly located on the edges of the tumor [11].

For example, it has been documented that the myofibroblast-like CAFs subpopulation was mostly located in areas that were adjacent to the tumor mass, whereas the pro-inflammatory CAFs subpopulation occupied a more distant position from the tumor mass in pancreatic ductal adenocarcinoma (PDAC) [40]. In agreement with the suggested similarity between CSCs and myofibroblasts-like CAFs, it has been described that this CAFs subpopulation is able to form spheroid structures [29], which is also a well-known property of CSCs.

**Fig. 1:**
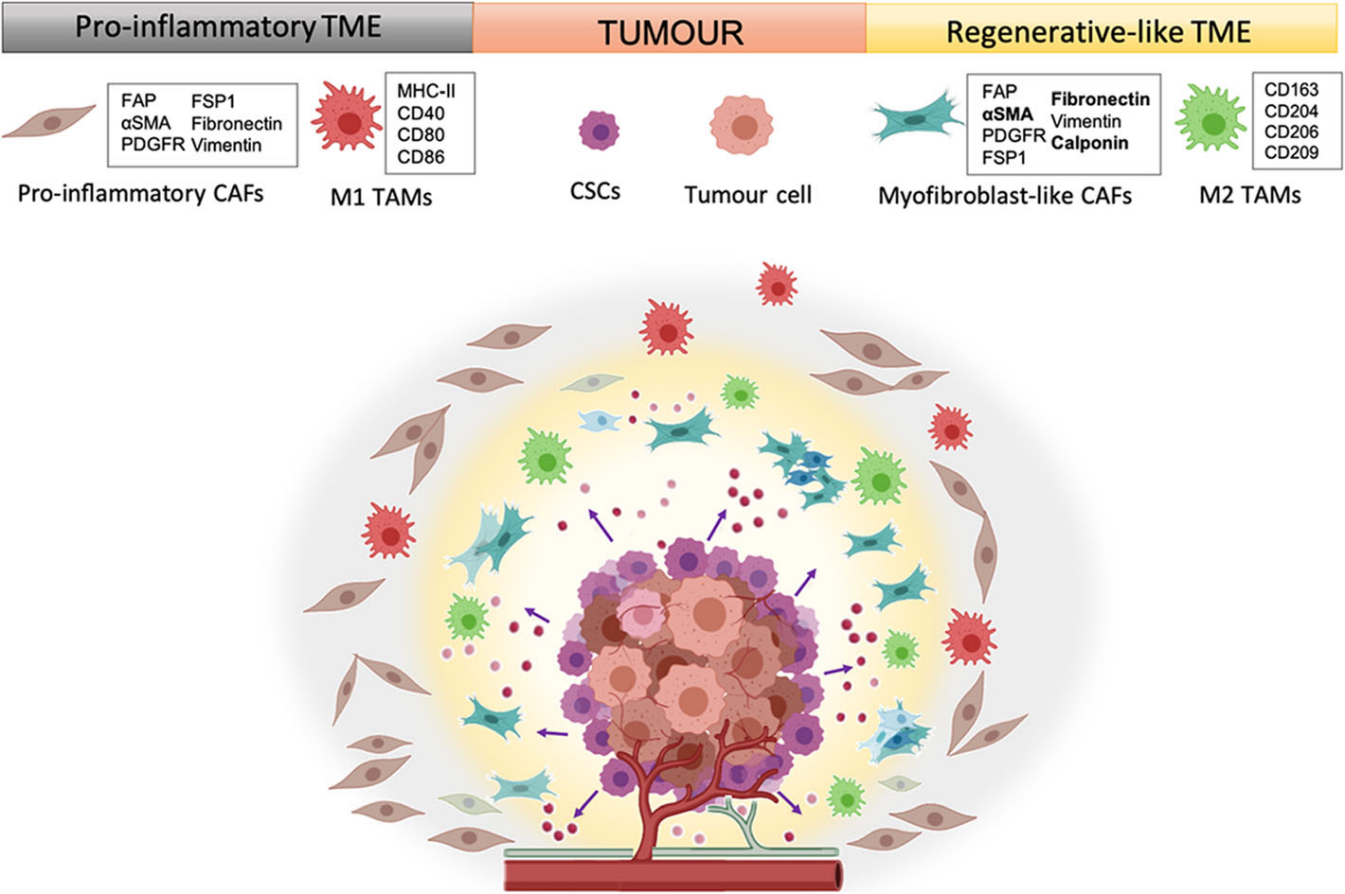
Sub-microenvironments within the TME of solid malignancies. TME is composed of an extensive vascular network, lymphatic vessels, soluble molecules, extracellular matrix and cells. Molecular factors secreted by cancerous cells, including CSCs, promote the corruption of healthy cells and the nature of the tumoral cells appears to condition the surrounding microenvironment. Based on this, it has been proposed two different sub-microenvironments: (i) a regenerative-like TME with a high accumulation of myofibroblast-like CAFs and tumor-supportive M2 TAMs located close to the CSCs in the tumor mass edges; and (ii) a pro-inflammatory TME with a high accumulation of both pro-inflammatory CAFs and tumor-suppressive M1 TAMs more distant from CSCs localization. Created with BioRender.com

Regarding to TAMs, it has been revealed that CD68+/CD163 + M2 TAMs progressively accumulated from the tumor nest and distant locations in the TME towards the invasive front of the tumor mass in cutaneous melanoma [41]. In another study in glioblastoma, the authors observed that a TME distant from the tumor mass mainly contained pro-inflammatory TAMs whereas the microenvironment nearer to the tumor mass mainly contained anti-inflammatory TAMs [42]. This fact is relevant since it has been reported that M2 macrophages may be related with an anti-inflammatory phenotype [43]. Considering the well-known relationship between myofibroblasts and wound healing along with the exposed relation between M2 macrophages and tissue repair/regeneration, it is tempting to hypothesize the existence of two sub-microenvironments within the TME of solid malignancies: (i) a regenerative-like TME with a high accumulation of myofibroblast-like CAFs and M2 TAMs, located close to the CSCs in the tumor mass edges and (ii) a pro-inflammatory TME with a high accumulation of both pro-inflammatory CAFs and M1 TAMs more distant from CSCs localization (Fig. 1).

It is important to clarify that the spatial organization of CSCs in the edges was noted to likely appear in an advanced stage of tumor development [11]. Therefore, the establishment of the two sub-microenvironments may also be observed in an advanced stage of tumor development but not in earlier stages (see following section). In summary, the expression-based and the functional-based cancerous cells heterogeneity along with the cell spatial organization of solid tumors, may reflect on the surrounding TME.

## Tumor microenvironment: a reflection of tumor molecular signature

It is widely accepted that the vast majority of solid tumors are able to promote the transformation of surrounding tumor-suppressive healthy fibroblasts into tumor-supportive CAFs [44]. Interestingly, this fact may sound like “transforming enemies into allies”, by “molecular corruption”. It has been reported that there is a variety of molecular factors secreted by cancerous cells which promote such corruption, with some examples being MMP7, PDGF, prograstin, sonic hedgehog, TGFB1, YAP [45], SDF1 [46], EGF [47] or FGF [48]. Considering CAFs as a consequence of the tumorigenic activity, it seems reasonable to suggest that the specific molecular signature of each malignancy may condition the molecular corruption of healthy precursor cells to generate CAFs with specific molecular profiles. Indeed, an association between the molecular factors that trigger the corruption of healthy fibroblasts and the final molecular profile of the CAFs can be detected. For instance, it has been shown that there is a strong relationship between the corruption of healthy fibroblasts by TGFβ into CAFs expressing αSMA [49], fibronectin or calponin [29]. In agreement, a correlation between different types of skin malignancies, such as cutaneous basal cell carcinoma, squamous cell carcinoma and malignant melanoma, and specific CAFs subtypes based on expression profiles has been documented [50]. Moreover, the existence of a similar specific association between CAFs subtypes and different tumor cell lines of the same malignancy type (MCF7 and MDA-MB-231 breast cancer cell lines) has been shown [51], supporting the idea about the reflection of the molecular signature of the tumor mass into the surrounding TME.

In addition, it is known that the molecular signature of the tumor changes or “evolves” during tumor progression. A good example of this event is the TGFβ pathway, which has been reported to be tumor-suppressive in earlier stages of tumor development and later become a tumor-supportive pathway in advanced tumor stages [52, 53]. Thus, those changes in the cancer molecular signature may explain the documented association between different CAFs subtypes (according to expression profiles) and distinct histological grades of breast cancer [51]. Interestingly, several studies have presented TGFβ pathway status as a key hallmark which differentiates the two functional CAFs subgroups stated earlier [9, 27, 54]. Furthermore, these studies also presented the myofibroblast-like CAFs subtype (also termed “TGFβ-responsive CAFs”) as CAFs in an advanced stage of corruption. According to these observations, it is important to note that αSMA, fibronectin and calponin are three myofibroblast-like CAFs representative biomarkers which are closely associated with the TGFβ corruption of CAFs. Additionally, Biffi and co-workers studied the molecular pathways of inflammatory CAFs and myofibroblast-like CAFs of pancreatic ductal adenocarcinoma [28]. They established that tumor cells-secreted IL1 promoted an inflammatory-like phenotype in CAFs via JAK/STAT signaling pathway, and that this event could be antagonized by the exposure of these inflammatory CAFs to TGFβ1. Such exposure triggered a switch from an inflammatory-like phenotype to a myofibroblast-like one in a dominant manner.

Tumors show different ratios between CAFs and TAMs. While pancreatic, breast and prostate cancers present high amount of CAFs in their TME, renal or ovarian tumors generally exhibit a less fibrotic TME [55]. Conversely, brain malignancies like gliomas are characterized by a large amount of TAMs in their TME [56]. Curiously, a similar event can be observed in other low-fibrotic malignancies such as ovarian cancer [57]. However, not all types of malignancies exhibit the same immune microenvironment since it has been established that there are six immune-based subtypes of malignancies according to total lymphocytic infiltrate, immune cell fractions, immune gene expression signatures, neoantigen prediction, viral RNA expression and somatic DNA alterations (wound healing, IFN-γ dominant, inflammatory, lymphocyte depleted, immunologically quiet, and TGFβ dominant) [58], reinforcing the idea that the molecular signature of each tumor exerts a specific influence on its environment. Importantly, Thorsson and co-workers documented an association between specific genetic mutations of tumor cells with some characteristics of the TME, for instance, the degree of leukocyte infiltration. In fact, it has been reported that specific mutations found in different brain tumors determine the final molecular profile of TAMs [59]. Consistent with this data, proteomics-based studies using mass cytometry have reported the existence of tumor-specific immune cell landscapes in different malignancies like renal cell carcinoma, lung cancer, colorectal adenocarcinoma, hepatocellular carcinoma, glioma, or melanoma [60].

**Fig. 2:**
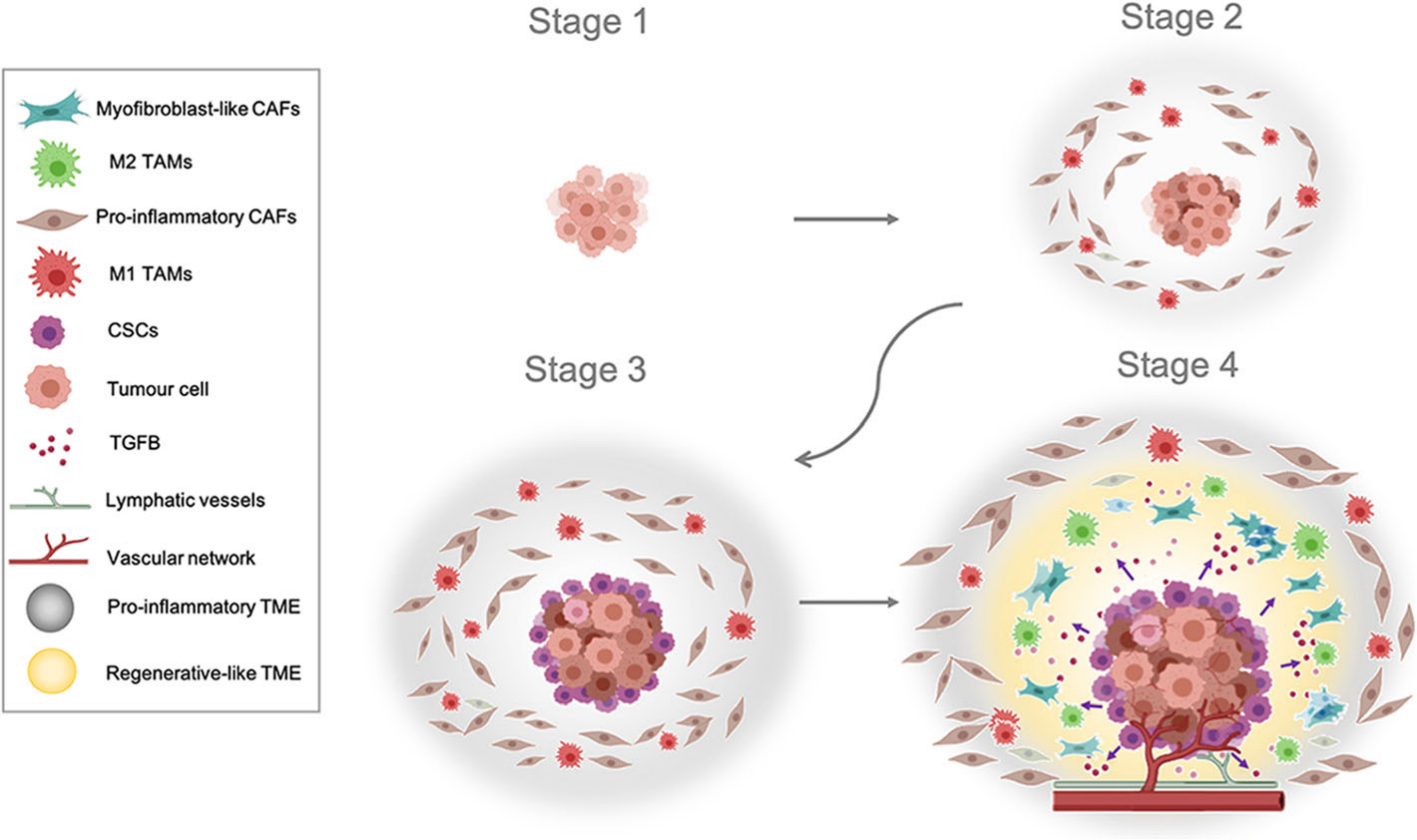
Hypothetical stages of primary solid tumors. Stage I: The tumor is small and has not spread to distant organs. Stage II: The tumor is larger and a pro-inflammatory TME appeared with a high accumulation of both pro-inflammatory CAFs and M1 TAMs. Stage III: high accumulation of pro-inflammatory CAFs and M1 TAMs, CSCs mainly in the edges of tumor mass start to appear. Stage IV: High appearance of CSCs in the edges of tumor mass, the molecular balance changes and promotes the generation of a regenerative-like microenvironment. This regenerative-like microenvironment prompts the accumulation of both myofibroblast-like CAFs subtype (also termed “TGFβ-responsive CAFs”) and tumor-supportive M2 TAMs polarized by TGFβ signalling. The cancer spreads to distant organs. Created with BioRender.com

In agreement with what has been described above regarding CAFs, there is also an “evolution” of immune cell phenotypes toward a more tumor-supportive role in advanced stages of tumor development [61]. It has been proposed that there is a tumor progression model in which a high M1/M2 TAM ratio is present in early stages whereas a low M1/M2 ratio is present in advanced stages [62, 63]. In agreement with these observations, we hypothesize the predominance of an inflammatory TME in the earlier stages of tumor development, characterized by a high accumulation of pro-inflammatory CAFs and/or pro-inflammatory M1 TAMs. The recruitment and polarization of M1 TAMs may be promoted by the secretion of a variety of molecular factors, for example, the pro-inflammatory cytokine IFNγ [64], by tumor cells and, interestingly, by activated fibroblasts (like pro-inflammatory CAFs) [65]. Next, the appearance of CSCs mainly in the edges of tumor mass in an advanced stage drastically changes the molecular balance and promotes the generation of a regenerative-like microenvironment just adjacent to the tumor mass edges. Such a regenerative-like sub-microenvironment may prompt a large accumulation of both myofibroblast-like CAFs and tumor-supportive M2 TAMs polarized by TGFβ signalling [66] (Fig. 2).

## Tumor cells force corruption of associated fibroblasts to work for them

Interestingly, it has been demonstrated that the transformation of healthy fibroblasts into CAFs is associated with changes in their energy metabolism, remarking on the switch from mitochondrial oxidative phosphorylation to aerobic glycolysis as the main source of energy. Such a switch is known as “Warburg effect” which mirrors the own cancer cell metabolism [67]. Notably, the so-called “aerobic glycolysis” is characterized by the prevalence of glycolysis over oxidative phosphorylation even in presence of sufficient oxygen. In the case of tumor cells, this switch could be explained by DNA mutations which lead to metabolic behaviour alterations. However, this is not the case for CAFs since it has been revealed that the corruption of stromal cells into CAFs by the master cytokine TGFβ relies on epigenetic mechanisms, but not on genetic mutations [68]. In agreement, CAFs seem to keep the genetic status of their healthy precursors [69]. Interestingly, it has been described that CSCs can differentiate into stromal cells like CAFs and TAMs [70]. So, a tumor mass can “re-educate” healthy fibroblasts or macrophages to become their allies or promote CSCs differentiation into those stromal cells. Therefore, the genetic alterations in TME cells found by some researchers could be associated with the cancerous origin of such stromal cells, but not by the corruption process. Remarkably, changes in the metabolic patterns of CAFs seem to not be related to covering their own energy needs but to serving tumor requirements. Indeed, it has been described that CAFs secrete the energy-rich metabolites obtained from the aerobic glycolysis in order to feed the mitochondrial oxidative phosphorylation of tumor cells (idea represented by a metabolic coupling model) [71]. This study focused on the so-called “reverse Warburg effect” that postulates the ability of tumors cells to enhance both glycolysis or oxidative phosphorylation according to their energy needs. In agreement with this fact, it has also been revealed that there is an almost unidirectional transference of mitochondria from CAFs to tumor cells [72]. Furthermore, the metabolic changes during CAFs corruption are not only related to the CAFs adaptation to the surrounding environmental conditions, but also lead to the CAFs self-destruction by autophagy [72, 73]. Thus, the intrinsic nature regarding the establishment of the TME, highlighting CAFs, can be interpreted as a “forced corruption” triggered by the continuous exposure of tumor surrounding healthy cells to tumor-derived factors. Indeed, considering the high energy requirement of tumor cells to maintain a high proliferative ratio and the hypoxia conditions of the vast majority of solid malignancies, the promotion of an aerobic glycolysis in CAFs by tumor cells may sound like a tumor order: “do not consume the residual oxygen…I need it”.

## Selective metastases: the distant reflection of primary TME

Metastatic growth is considered as the main cause of cancer-related death although it is not yet a well-understood process [12]. It is known that a chain of changes in gene expression profiles directs primary tumor cells to become metastatic cells. This genetic program is known as “the core metastatic gene expression program” and is mainly shared by all types of metastatic cells. These orchestrated events that lead to metastasis include the induction of vascularization and tissue remodelling, the weakening of the physical interactions between primary tumor cells and the extracellular matrix, the alteration of ions homeostasis, and the induction of oxidative metabolism [74]. Furthermore, recent evidence gives increased relevance to epigenetic alterations, rather than metastasis-promoting DNA mutations, as the molecular mechanisms underlying the primary tumor cells progression toward a pro-metastatic phenotype. As an example, the acquisition of a migratory and pro-metastatic phenotype by pancreatic tumor cells through the epigenetic silencing of GATA6, promoted by the upregulation of miR-27a has been reported [75]. Interestingly, non-genomic alterations that end in inducing a metastatic phenotype are documented to rely on post translational modifications. In this sense, acetylation [76] or sialylation [77] of protein domains belonging to relevant cellular pathways like the TGFβ pathway, which undergo key changes during tumor progression, can be highlighted.

Moreover, it is interesting to note some links between metastatic growth and the primary tumor. First, it has been recently reported that the overexpression of L1CAM relates colorectal cancer metastases with a regenerative-like pattern [78]. Considering that Ganesh and colleagues noted that the regenerative-like profile was not found at the origins of the primary tumor, and taking into account that L1CAM has been documented to be a CSCs marker [79], it seems reasonable to assume that the metastatic growth may be closely related to the previous appearance of CSCs along with the regenerative-like sub-microenvironment in an advanced stage of the primary tumor development. Furthermore, it has been shown that once metastatic cells colonize the target organ, they adapt their gene expression profiles toward those characteristics of the metastatic tissue and partially loss those characteristics of the organ of origin [80]. Nevertheless, it is important to highlight that metastatic tumors may still keep some expression profiles characteristic of the primary tumors as a distant reflection of their origins. Indeed, it has been revealed that there is a possibility of determining the location of an undetected primary tumor by analyzing the gene expression patterns of the detected metastasis (using, for instance, the Iowa Net Site of Origin Classifier algorithm based on different biomarkers expression patterns) [81]. Thus, the metastasis-related gene expression profiles may be comprised of both primary tumor-specific patterns and target site-specific ones.

Additionally, a promising link between the primary tumor molecular signature and the future site of metastasis could be established. In this respect, it has been observed that breast and prostate cancers usually metastasize in bone [13], pancreatic cancer usually metastasizes in the liver [82], or lung metastases of salivary adenoid cystic carcinomas [83]. One possible explanation for such events could be the existence of a “default molecular plan” in each primary tumor which pushes them to colonize specific distant organs in an advanced stage. In accord to this idea, CTGF-RUNX2-RANKL as a molecular axis by which primary MDA-MB-231 triple negative breast cancer cells and PC3 prostate cancer cells could develop future selective bone metastases has been presented [13]. From an alternative point of view, it has been noted that the primary TME may also play a key role in guiding future metastases. Briefly, it has been suggested that certain primary TME-derived cells, like CAFs, could establish specific molecular balances resembling those existing in the future metastatic organ. Tumor cells will then be selected depending on their gene expression patterns to outgrowth in distant organs with similar molecular balances of the surrounding primary TME [84].

On the other hand, the formation of a pre-metastatic niche may start in the earlier stages of primary tumor development like the local primary TME [82], promoted by tumor cells-derived soluble factors and extracellular vesicles [85]. Therefore, it may be considered as a distant primary TME with TAMs and CAFs as key components [86, 87].

It is known that cells from the primary tumor mass can establish pre-metastatic niches which support circulating tumor cells (CTCs) nesting and future metastatic outgrowth [14]. Those distal niches commonly exhibit a permeabilized vasculature and an enrichment of E-selectin on the luminal surface of its endothelium, which triggers the CTCs adhesion and extravasation [88]. Other studies have shown a cytokine and chemokine gradient governing CTCs homing to the pre-metastatic niche [89]. Therefore, this data may imply the site-selective formation of the pre-metastatic niche as a key event for the site-specific metastatic outgrowth. In fact, it has been observed that the inoculation of Lewis lung carcinoma cells, which only metastasize in the lung, into mice pre-treated with a melanoma-conditioned medium generated metastases not only in the lung but also in the oviducts, spleen, intestine and kidneys (common sites of melanoma metastases) [90]. Thus, melanoma secreted factors are conditioning the appearance of “non-conventional” pre-metastatic niches.

Cells from the primary tumor mass encapsulate molecular factors inside tumor-derived exosomes and direct them toward the pre-metastatic niche by coating their membranes with targeting signals. For instance, it has been shown that breast and pancreatic cancers-derived exosomes loaded with α6-β4 integrin on their surface are directed to pulmonary fibroblasts whereas exosomes coated with α6-β1 are guided and incorporated into pulmonary epithelial cells (both promoting lung metastases). In addition, exosomes with αv-β5 integrin are incorporated by Kupffer cells provoking liver metastases [15]. Besides integrins, tetraspanins have been described as important factors involved in the selective uptake of exosomes. For instance, tSPaN8-a4 exosomes are selectively uptaken by endothelial cells while TSPAN8-a6b4 exosomes are more effectively incorporated by fibroblasts [91]. Additionally, the relevance of protein complexes on the exosome surface, like tetraspanins-integrins complexes, rather than individual molecules in the selective exosome uptake process has been reported [92].

**Fig. 3:**
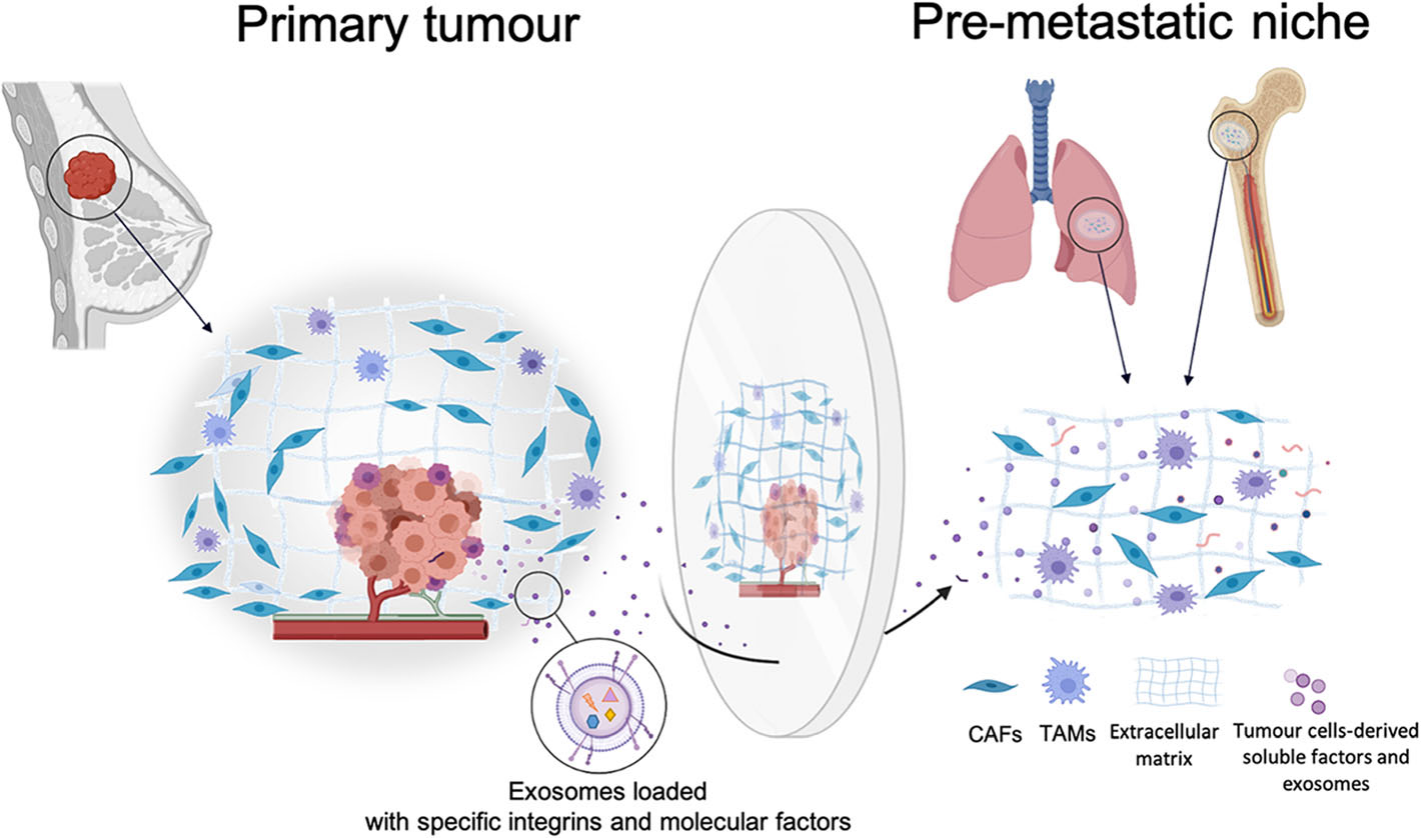
Formation of a pre-metastatic niche. The formation of a pre-metastatic niche (with both CAFs and TAMs) occurs before metastatic growth and it is promoted by primary tumor-derived soluble factors and exosomes. The specific integrins patterns on the exosome surface may condition the specific cellular targets of such exosomes. Therefore, the molecular signature of the pre-metastatic niche and its site-selective location may be considered as a reflection of the primary tumor molecular signature. Created with BioRender.com

Hence, the location of the pre-metastatic niche may be determined by the surface molecular configuration of primary tumor-derived exosomes thus being a direct reflection of primary tumor molecular signature (Fig. 3). Moreover, metastatic growth may be related to the regenerative TME; in other words, metastatic location and growth may be considered as a double reflection of the primary tumor molecular signature that changes during tumor progression. Indeed, the existence of different exosome markers associated with certain stages of tumor progression has already been documented [93], thus it may determine new metastatic locations.

## Diagnostic and therapeutic considerations

The heterogeneous and complex nature of the tumor surrounding tissue is a main factor that complicates the implementation of an effective treatment in many cancer patients. Due to the close relationship between the primary tumor cells gene expression profile, the primary TME, and the pre-metastatic niche location, the improvement of common strategies to analyse solid and liquid biopsies is key to increase an accurate prognosis and treatment of cancer. For instance, post-translational modifications like sialylation or acetylation of certain key pathway components, like the TGFβ pathway, should be determined in cancer cells in order to study tumor development. Indeed, the information obtained from post-translational modifications analyses may be valuable data to complement the information obtained from gene expression profiles analyses in order to establish the tumor progression timepoint [60, 77]. However, solid biopsy analyses commonly face the risk of being non-representative samples as a consequence of the high intratumor heterogeneity characteristic of many solid malignancies [94]. Thus, it may be relevant to closely study tumor edges biopsies searching for CSCs markers. In fact, the presence of a large proportion of CSCs might indicate an advanced stage of tumor progression, higher risk of resistance to radio- and chemotherapy, increased risk of metastasis and recurrence after treatment [95]. Unfortunately, the huge range of CSCs-associated biomarkers between different types of tumors and even between different patients with the same tumor type, and the small tumor size at diagnosis time may limit such a strategy [96].

For these reasons, a systematic analysis of the TME just adjacent to the tumor mass may overcome this limitation, even more in the case of certain malignancies, like pancreatic tumors, characterized by small tumor sizes compared with its surrounding TME [97]. For instance, it may seem interesting to establish the proportion of myofibroblasts-like CAFs compared with the total amount of CAFs [29] and/or to analyze TAMs establishing the proportion of M2/M1 [63] to determine the presence of the regenerative sub-microenvironment and thus predict the presence of CSCs. Interestingly, both CAFs- and TAMs-associated biomarkers may represent a less heterogeneous group compared with CSCs-associated markers, so the analysis of TME-derived cells may have a higher standardization potential in clinical practice.

Furthermore, in recent years the characterization and analysis of exosomes produced by tumor cells and TME-derived cells have become a priority based in the accepted relationship of these vesicles with the pre-metastatic niche formation [98, 99]. An appealing strategy may rely on the development of an “exosomes trap” to intercept messages sent from the primary tumor to distant tissues. The significant development in the past decade around extracellular vesicles technology [100] suggests the possibility of using functionalized nanoparticles coated with specific ligands of tumor exosome-related integrins and/or tetraspanins to bind secreted exosomes. Such an “exosome trap” would competitively neutralize them in a similar manner like that in which it has already been proposed to use cell membrane-coated nanoparticles as detoxification agents to neutralize organophosphates [101]. Moreover, it has been shown that CAF-derived extracellular vesicles are also able to promote the formation of a pre-metastatic niche in a distant secondary organ like the lungs [99], so the complexity of the exosome-based analysis may be even higher including primary TME-derived extracellular vesicles.

Nevertheless, it is important to consider that, although exosomes have generated great excitement due to their potential as cancer biomarkers, there are still certainly many technical difficulties, such as the improvement of high-throughput methodologies for their enrichment, to reach a standard and effective procedure of use in hospitals.

It is an obvious statement that metastatic cells need a place to nest, so predicting where a pre-metastatic niche is likely to be established by the acute characterization of tumor-derived exosomes may lead to its early diagnosis before its colonization by metastatic cells. This event would permit the targeting of the cells that have been corrupted to prepare the appropriate pre-metastatic niche. The basis of the “re-education” therapies consists of switching tumor-supportive stromal cells into tumor-suppressive ones. Thus, for instance, reversing CAFs activation status [102] and/or promoting the switch of TAMs from M2 to a M1 phenotype (termed as “repolarization therapy”) [57] could be an effective approach in impairing the pre-metastatic niche establishment and the metastatic process. Furthermore, it may also be a complementary therapeutic option to the already established anticancer treatments against the primary tumor. However, there is a key difference between the metastatic and primary tumors: whereas metastatic growth is characterized by the generation of the TME (pre-metastatic niche) before the metastatic cells colonization and growth, the primary tumor growth is characterized by the appearance of cancerous cells and then the primary TME corruption by them. Thus, the use of the re-education therapies after primary tumor removal may be an interesting approach in order to avoid the “re-corruption” of primary TME considering the direct and constitutive “nature” of the corrupting signals derived from the primary cancer cells.

In addition, it is interesting to remark that cellular pathways are regulated in a similar manner among tumor cells and stromal cells from the surrounding TME, so one could suggest using a single drug to treat both tumor mass and the TME simultaneously. Indeed, this idea has already been reflected in a recent study in which targeting the IL1/NFKB pathway might disrupt both pancreatic ductal adenocarcinoma development and associated CAFs [103]. In agreement, it is important to remark that the majority of pancreatic ductal adenocarcinomas exhibit an overexpression of NFKB pathway [104], extensive fibrosis and up-expression of IL1, which promotes the generation of inflammatory CAFs by pancreatic tumor cells.

## Conclusions

In conclusion,TME and metastasis can seem like independent and chaotic processes but they have an intrinsic order, which can be used to improve our clinical management of cancer. Furthermore, the prediction of the risk of a metastatic growth and the pre-metastatic niche location has relevant clinical implications and may be achieved by an accurate characterization of adjacent to tumor mass TME biopsies and primary tumor-derived exosomes, respectively.

## Data Availability

Not applicable.

## Abbreviations

TME: Tumor microenvironment
CSCs: Cancer stem-like cells
CAFs: Cancer-associated fibroblasts
TAMs: Tumor-associated macrophages
MSCs: Mesenchymal stem cells
MARCO: Macrophage receptor with collagenous structure
PDAC: Pancreatic ductal adenocarcinoma
CTCs: Circulating tumor cells
